# Residue Behavior, Processing Factors, and Dietary Exposure of Buprofezin and Etofenprox During Rice Processing

**DOI:** 10.3390/foods15213846

**Published:** 2026-10-27

**Authors:** Dongju Kim, Seunghyeon Jo, Hyeonwoo Shin, Youngjin Ham, Junyoung Kim, Eunbeen Oh, Mihyun Cho, Moohyeog Im, Keesung Kyung

**Affiliations:** 1 Department of Environmental and Biological Chemistry, College of Agriculture, Life and Environment Sciences, Chungbuk National University, Cheongju 28644, Republic of Korea; kimdj6746@naver.com; 2 Republic of Korea Institute of Toxicology, Jeongeup 56212, Republic of Korea; seunghyeon.jo@kitox.re.kr; 3 NongHyup Chemical, Okcheon 29008, Republic of Korea; hyeonwoo@nhchemical.com; 4 Residue Study Division, AB Solution, Hwaseong 18514, Republic of Korea; youngjin0223@naver.com; 5 Republic of Korea Conformity Laboratories, Incheon 21999, Republic of Korea; tim8518@kcl.re.kr; 6 Dongbang Agro Corporation, Buyeo 33216, Republic of Korea; eboh01@dbagro.co.kr; 7 Department of Food Engineering, College of Engineering, Daegu University, Gyeongsan 38453, Republic of Korea; c_mh97@naver.com (M.C.); imh0119@daegu.ac.kr (M.I.)

**Keywords:** dietary exposure, food safety, pesticide residues, processing factor, rice processing

## Abstract

Rice processing can substantially alter pesticide residues before consumption. This study evaluated the residue behavior of buprofezin and etofenprox during rice processing, determined their processing factors (PFs), and assessed dietary exposure through rice and processed rice products. To ensure quantifiable residues throughout the study, both pesticides were applied at three times the recommended Korean good agricultural practice (GAP) rate. Relative to whole grain, buprofezin and etofenprox residues decreased by 91.9% and 96.8% in husked rice, respectively, with the greatest overall reductions observed in polished rice (99.0% and 99.5%, respectively). In contrast, higher residue concentrations were observed in hulls, with PFs of 6.45 for buprofezin and 3.60 for etofenprox. Cooking further reduced residues remaining in husked rice, whereas both pesticides were below the limit of quantification (LOQ) in cooked polished rice and rice cake. Despite application at three times the recommended GAP rate, estimated chronic dietary exposure was low, with maximum percentage of acceptable daily intake (%ADI) values of 1.303% for buprofezin and 0.261% for etofenprox. These findings indicate that milling was the predominant processing step reducing pesticide residues in edible rice fractions, and cooking provided an additional reduction before consumption.

## 1. Introduction

The use of pesticides is an integral component of crop production, helping to limit losses caused by pests and diseases and maintain crop yield and quality [1,2]. However, pesticide residues may remain in agricultural commodities and contribute to human dietary exposure [3]. Residue levels in crops are influenced by application conditions, the physicochemical properties of active ingredients, environmental conditions, crop growth stage, and the interval between pesticide application and harvest [4,5]. Crop characteristics may also affect pesticide deposition; for example, leaf surface characteristics have been shown to influence the deposition and distribution of spray droplets on rice plants [6].

Residue concentrations measured in raw agricultural commodities do not necessarily represent the levels present in foods as consumed because agricultural products commonly undergo various processing steps before consumption. Washing, peeling, milling, soaking, heating, and cooking can alter pesticide residue concentrations [7,8,9]. Processing may reduce residues through physical removal, dissolution, or degradation, whereas higher residue concentrations may be observed in certain processing fractions [9,10,11]. The nature and magnitude of these changes depend on processing conditions and the physicochemical properties of the pesticides [9,11].

The processing factor (PF) quantitatively describes the effect of food processing on pesticide residue concentrations and is generally calculated as the residue concentration in a processed product divided by that in the corresponding starting material [12]. PF values below 1 reflect a decrease in residue concentration after processing, whereas values above 1 indicate a higher residue concentration in the processed fraction [12]. Accordingly, PFs provide useful information for characterizing the effects of food processing on pesticide residues and refining estimates of dietary exposure [13]. Previous studies on cereal processing have shown that milling can substantially reduce pesticide residues in edible grain fractions, while higher residue concentrations may occur in by-products such as hulls or bran [10,14,15].

Rice typically undergoes sequential removal of the outer grain layers before consumption. Dehulling separates the hull to produce husked rice, and subsequent polishing removes additional outer fractions to yield polished rice. However, differences in rice varieties and the degree of milling may influence pesticide residue distribution and removal during processing [7,15]. Therefore, the findings obtained from a single rice variety under specific milling conditions may not be directly generalizable to other rice varieties or processing conditions. The edible grain may then undergo additional processing, including washing, soaking, cooking, or preparation into products such as rice cake. Because pesticide residues can be unevenly distributed among grain fractions, concentrations measured in harvested whole grain may not accurately reflect those present in rice and rice products as consumed. Furthermore, cooking and other subsequent processing steps can further modify residue concentrations [15,16].

Buprofezin and etofenprox are insecticides used to control agricultural pests, including those affecting rice production. Buprofezin is an insect growth regulator that inhibits chitin biosynthesis and interferes with insect molting (IRAC Group 16), whereas etofenprox is a pyrethroid-like ether insecticide that disrupts insect nerve function by modulating voltage-gated sodium channels (IRAC Group 3A) [17]. Both compounds exhibit low water solubility and high lipophilicity, with etofenprox being more hydrophobic than buprofezin [18,19]. These physicochemical properties may influence their distribution among rice grain fractions and their behavior during processing. In addition to their agricultural applications, potential concerns regarding human and animal health and environmental effects warrant consideration. Regulatory assessments have identified endocrine-disrupting properties relevant to human health and reproductive risks to wild mammals for buprofezin [20], while etofenprox has been reported to exhibit toxicity to aquatic organisms and bees [19]. Furthermore, dietary exposure to pesticide residues requires consideration of both toxicological properties and actual residue concentrations in foods [20,21]. Therefore, investigating the processing behavior of buprofezin and etofenprox in rice is important for understanding residue distribution during processing and assessing potential dietary exposure through rice and its processed products.

Previous studies have investigated pesticide residue changes during rice processing, but the extent of residue reduction varies depending on the pesticide and processing conditions. Barad et al. [22] evaluated the effects of different household cooking methods on pesticide residues in rice, while Kim et al. [23] investigated the processing factors of flubendiamide and tebufenozide during rice milling, cooking, and rice cake preparation. The recent update of the European Union processing factor database further highlights the importance of reliable pesticide- and commodity-specific processing data for dietary exposure assessment [24]. Nevertheless, additional data are needed to characterize the processing behavior of buprofezin and to complement existing findings on etofenprox across different rice fractions and processed products. Therefore, the present study provides compound-specific experimental data on residue distribution, processing factors, and estimated chronic dietary exposure under defined field and processing conditions.

Accordingly, this study evaluated the residue behavior of buprofezin and etofenprox throughout sequential rice processing stages. Residue concentrations were determined in whole grain, husked rice, polished rice, hulls, inner chaff, rice straw, cooked rice, and rice cake, and PFs were calculated to quantify changes during milling and cooking. Dietary exposure to both pesticides was also estimated using food-consumption data and acceptable daily intake (ADI) values. By integrating residue measurements across successive processing stages with PFs and dietary exposure estimates, this study provides data relevant to assessing pesticide residues in rice and rice products as consumed.

## 2. Materials and Methods

### 2.1. Field Trials

Samkwang rice (*Oryza sativa* L.) was used for the processing study conducted in Anseong, Gyeonggi Province, Republic of Korea. This study was designed as a controlled field-based processing experiment rather than a market-based pesticide residue monitoring survey. Each pesticide treatment consisted of three independent field plots, and rice harvested from each plot was processed and analyzed separately without pooling samples across replicates. The untreated control plot covered 80.0 m^2^ (2.0 × 40.0 m), whereas an area of 144 m^2^ (2.0 × 72.0 m) was assigned to each pesticide treatment. Each treatment consisted of three replicate plots. The commercial formulations used in the study were Onnuri, a wettable powder (WP) containing 12% buprofezin and 5% tebufenozide, and Bisangtan, an emulsifiable concentrate (EC) containing 10% etofenprox and 4% tebufenozide (both supplied by KYUNG NONG Corporation, Seoul, Republic of Korea).

Buprofezin and etofenprox were applied to rice plants using a backpack sprayer (MSB1500Li, Maruyama Mfg. Co., Inc., Tokyo, Japan). The final applications of buprofezin and etofenprox were made 7 and 14 days before harvest, respectively, according to their respective pre-harvest intervals. Each pesticide was applied three times at 10-day intervals. For the processing study, the target application rates were set at three times the recommended Korean good agricultural practice (GAP) rates to obtain quantifiable residues throughout the processing stages, consistent with OECD Test Guideline No. 508 [25]. The nominal threefold GAP rates were 0.576 kg a.i./ha for buprofezin and 0.480 kg a.i./ha for etofenprox. The actual application rates ranged from 0.666 to 0.687 kg a.i./ha for buprofezin and from 0.572 to 0.585 kg a.i./ha for etofenprox, corresponding to deviations of +15.6% to +19.3% and +19.2% to +21.9% from the respective target rates. These deviations were within the ±25% range used by FAO/JMPR when assessing the comparability of application rates in supervised residue trials under otherwise comparable conditions [26]. The target and actual application rates are presented in Table 1.

At harvest, 71.5–84.2 kg of whole grain was collected from the buprofezin-treated plots and 79.9–81.9 kg from the etofenprox-treated plots, whereas 40.2–42.2 kg was collected from the untreated control plots. The harvested whole-grain samples were transported within 24 h and dried in a plastic greenhouse for 7 days until the moisture content was below 15%. After drying, the grain was processed to obtain husked rice, polished rice, hulls, pressure-cooked and electric-cooked husked rice, pressure-cooked and electric-cooked polished rice, and rice cake. All processed samples were individually sealed in polyethylene bags and stored at −20 °C until residue analysis.

### 2.2. Processing Procedures and Sample Preparation

**Figure 1:**
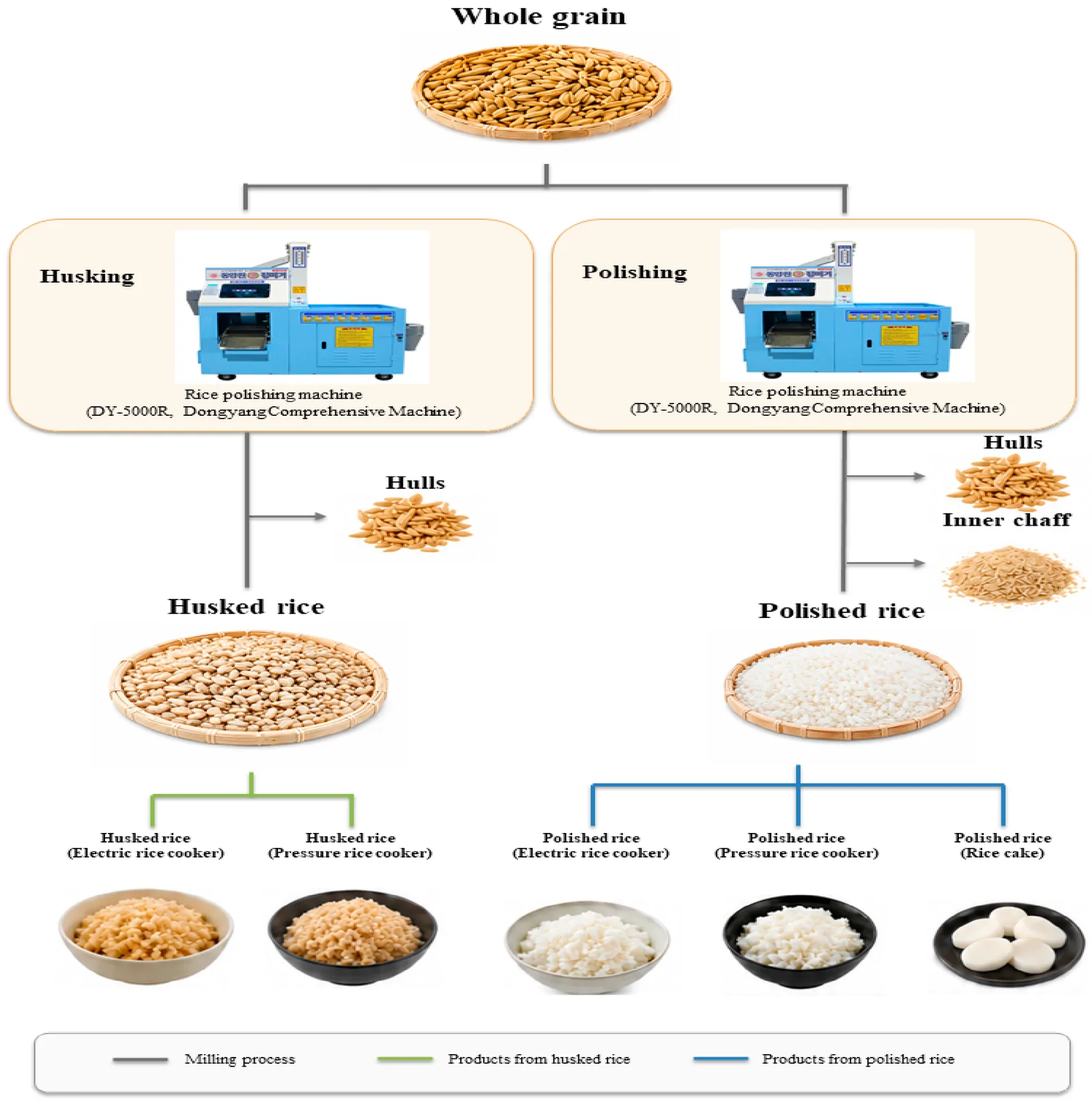
Processing scheme of whole grain into husked rice, polished rice, and their processed products.

Dried whole-grain samples from each of the three independent field replicates were separately divided into portions for milling and subsequent food-processing experiments, without pooling samples across replicates. Milling was performed using a rice milling machine (DY-5000R, Dongyang Comprehensive Machine, Yeongcheon, Republic of Korea). Husked rice was obtained by removing the hull, and polished rice was produced by further removal of the outer grain layers. Hulls and inner chaff generated during milling were collected separately for residue analysis (Figure 1).

For the cooking experiments, husked and polished rice were washed three times using water equivalent to 1.5 times the weight of the rice. For pressure cooking (CRP-HD1010FI, Cuckoo Electronics Co., Ltd., Yangsan, Republic of Korea), the washed rice was soaked for 30 min and drained, after which fresh water was added at a rice-to-water ratio of 1:1.5 (*w*/*w*). Husked and polished rice were then cooked for 30 and 20 min, respectively. For electric cooking (SB-56RC, Kitchen Art, Incheon, Republic of Korea), the same washing and soaking procedures were followed, and husked and polished rice were cooked for 30 and 40 min, respectively.

Rice cake was prepared from 1.7 kg of polished rice. The rice was washed three times, soaked for 12 h, and drained, after which 1% salt was added. The soaked rice was ground (Duksan Food Co. Ltd., Chungbuk, Republic of Korea), passed through a 20-mesh sieve, and homogenized. Water and sugar were each added at 10%, and the resulting mixture was kneaded and steamed at 100 °C for 20 min (HY-2009-A, Hanyang Metal, Ulsan, Republic of Korea). The rice cake was then cooled to room temperature. Samples collected at each processing stage were individually sealed in polyethylene (PE) bags and stored at −20 °C until residue analysis.

### 2.3. Reagents and Materials

Analytical standards of buprofezin (99.0% purity) and etofenprox (99.1% purity) were obtained from Sigma-Aldrich (St. Louis, MO, USA). HPLC-grade acetonitrile, used for the preparation of standard solutions, sample extraction, and chromatographic analysis, was purchased from Burdick & Jackson (Muskegon, MI, USA). HPLC-grade water was used for preparation of the LC mobile phase, and formic acid (>98%) was obtained from Samchun Chemicals (Seoul, Republic of Korea). QuEChERS extraction packets (roQ™ QuEChERS, Phenomenex, Torrance, CA, USA) were used for sample extraction. Sample extracts were centrifuged using a Combi-514R centrifuge (Hanil Scientific Inc., Gimpo, Republic of Korea), and homogenization and extraction were performed using a 2010 Geno/Grinder^®^ (SPEX SamplePrep, Metuchen, NJ, USA).

### 2.4. Sample Preparation

For each pesticide treatment, samples collected from three independent field replicate plots were processed separately without pooling. Each sample was subjected to a single extraction, and each resulting extract was analyzed by LC–MS/MS using a single instrumental injection. The mean residue concentrations and standard deviations were calculated from the measurements obtained from the three independent field replicates (*n* = 3), rather than from repeated extractions or instrumental injections of the same sample.

#### 2.4.1. Extraction

##### Whole Grain, Husked Rice, Polished Rice, Cooked Husked Rice and Polished Rice and Rice Cake

**Figure 2:**
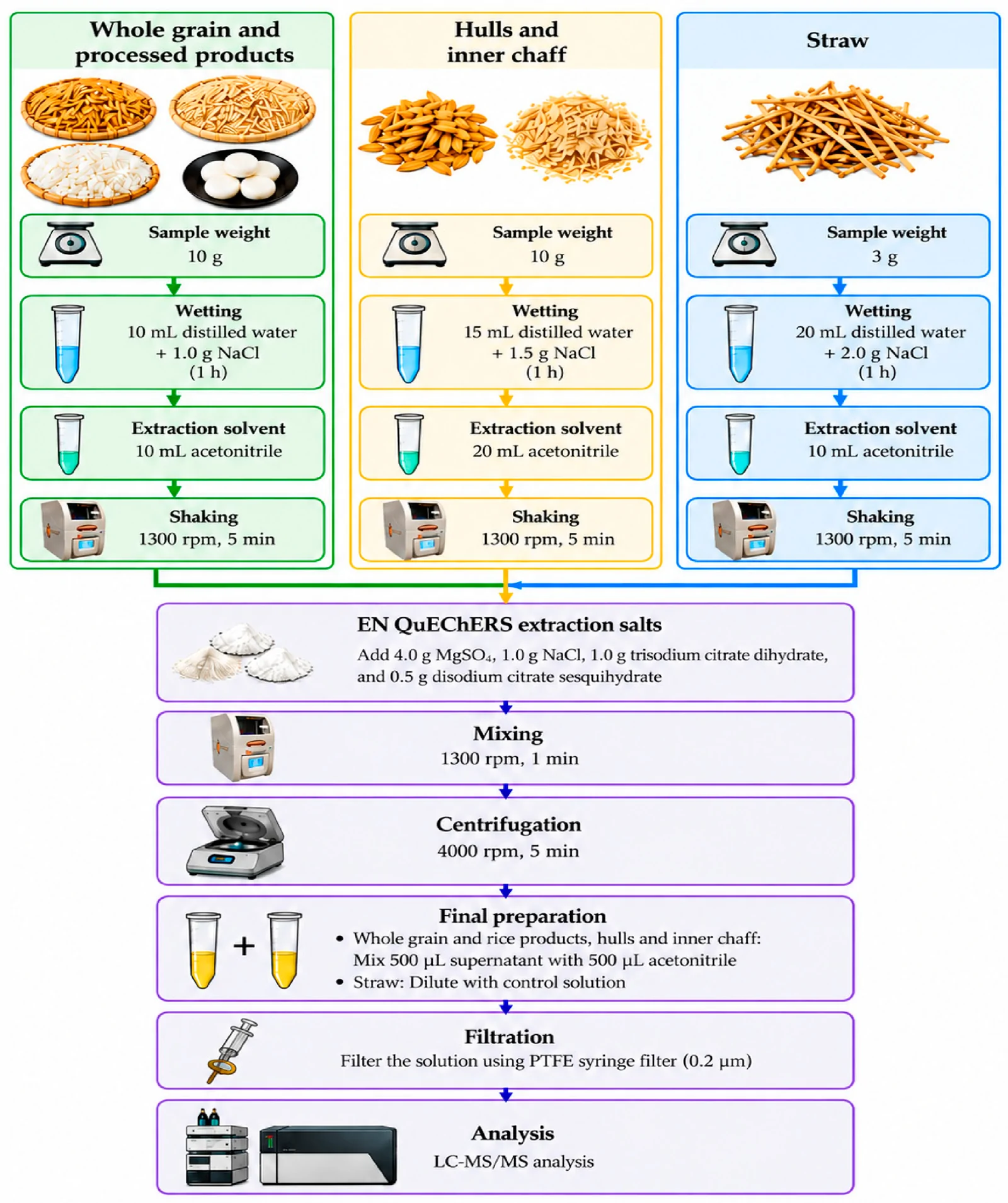
Sample preparation and QuEChERS extraction procedure for the analysis of buprofezin and etofenprox residues in rice, its processed products, and straw.

A 10 g portion of each sample was weighed into a 50 mL conical tube and wetted with 10 mL of distilled water containing 1 g of NaCl for 1 h. Subsequently, 10 mL of acetonitrile was added, and the tube was capped and shaken at 1300 rpm for 5 min. A QuEChERS salt mixture consisting of 4 g of MgSO_4_, 1 g of NaCl, 1 g of trisodium citrate dihydrate, and 0.5 g of disodium citrate sesquihydrate was then added. The tube was shaken again at 1300 rpm for 1 min and centrifuged at 4000 rpm for 5 min. An aliquot (500 μL) of the supernatant was mixed with 500 μL of acetonitrile, and the resulting solution was filtered through a 0.2 μm PTFE membrane filter (Phenomenex, Torrance, CA, USA) prior to LC–MS/MS analysis. The overall sample preparation and QuEChERS extraction procedures are summarized in Figure 2.

##### Hulls and Inner Chaff

A 10 g portion of each hull and inner chaff sample was weighed into a 50 mL conical tube and wetted with 15 mL of distilled water containing 1.5 g of NaCl for 1 h. Subsequently, 20 mL of acetonitrile was added, and the tube was capped and shaken at 1300 rpm for 5 min. A QuEChERS salt mixture consisting of 4 g of MgSO_4_, 1 g of NaCl, 1 g of trisodium citrate dihydrate, and 0.5 g of disodium citrate sesquihydrate was then added. The tube was shaken again at 1300 rpm for 1 min and centrifuged at 4000 rpm for 5 min. An aliquot (500 μL) of the supernatant was mixed with 500 μL of acetonitrile, and the resulting solution was filtered through a 0.2 μm PTFE membrane filter (Phenomenex, Torrance, CA, USA) prior to LC–MS/MS analysis.

##### Straw

A 3 g portion of the straw sample was weighed into a 50 mL conical tube and wetted with 20 mL of distilled water containing 2.0 g of NaCl for 1 h. Subsequently, 10 mL of acetonitrile was added, and the tube was capped and shaken at 1300 rpm for 5 min. A QuEChERS salt mixture consisting of 4 g of MgSO_4_, 1 g of NaCl, 1 g of trisodium citrate dihydrate, and 0.5 g of disodium citrate sesquihydrate was then added. The tube was shaken again at 1300 rpm for 1 min and centrifuged at 4000 rpm for 5 min. Following centrifugation, the supernatant was collected and filtered through a 0.2 μm PTFE membrane filter (Phenomenex, Torrance, CA, USA) prior to LC–MS/MS analysis. When dilution was required, the sample extract was diluted using a blank straw matrix extract. Matrix-matched calibration standards were prepared using extracts obtained from untreated straw samples.

### 2.5. Instrumental Analysis

Buprofezin was analyzed using an Acquity UHPLC H-Class system coupled with a Xevo TQD triple-quadrupole mass spectrometer (Waters Corporation, Milford, MA, USA) equipped with an Acquity UHPLC BEH C18 column (2.1 × 100 mm, 1.7 μm; Waters Corporation, Milford, MA, USA). Isocratic elution was performed using HPLC-grade water containing 0.1% formic acid and acetonitrile containing 0.1% formic acid (10:90, *v*/*v*). The flow rate was 0.3 mL/min, and the injection volume was 1 μL. Mass spectrometric detection was performed using an electrospray ionization (ESI) source in positive-ion mode. The MS conditions were as follows: capillary voltage, 3.0 kV; source temperature, 150 °C; cone gas flow, 55 L/h; desolvation temperature, 380 °C; and desolvation gas flow, 550 L/h. For multiple reaction monitoring (MRM) analysis of buprofezin, the parent ion was set at mass to charge ratio; *m*/*z* 306.2 with a cone voltage (CV) of 20 V. The product ions used for quantification and confirmation were *m*/*z* 201.3 and 116.2, with collision energies (CEs) of 15 and 20 V, respectively.

Etofenprox was analyzed using a Nexera X3 system coupled with an LCMS-8050 triple-quadrupole mass spectrometer (Shimadzu Corporation, Kyoto, Japan) equipped with a Phenomenex Kinetex^®^ C18 column (2.1 × 150 mm, 2.6 μm; Phenomenex, Torrance, CA, USA). Isocratic elution was performed using HPLC-grade water containing 0.1% formic acid and acetonitrile containing 0.1% formic acid (20:80, *v*/*v*). The flow rate was 0.3 mL/min, and the injection volume was 1 μL. Mass spectrometric detection was performed using an ESI source in positive-ion mode. The MS conditions were as follows: spray voltage, 4.0 kV; interface temperature, 150 °C; heat block temperature, 400 °C; DL temperature, 250 °C; drying gas flow, 10.0 L/min; heating gas flow, 10.0 L/min; and nebulizing gas flow, 3.0 L/min. For MRM analysis, the precursor ion of etofenprox was *m*/*z* 394.1, and the product ions used for quantification and confirmation were *m*/*z* 177.1 and 134.9, with CEs of −17.0 and −25.0 V, respectively. Separate LC–MS/MS systems were used for buprofezin and etofenprox to improve analytical throughput and reduce the overall analysis time. The suitability of each analytical method was independently evaluated through method validation, as described in Section 2.6.

### 2.6. Method Validation

The analytical methods for buprofezin and etofenprox were validated in accordance with the Codex Guidelines on Performance Criteria for Methods of Analysis for the Determination of Pesticide Residues in Food and Feed (CXG 90-2017) [27]. Method performance was evaluated in terms of linearity, limit of quantification (LOQ), accuracy, and precision. The LOQ was established at 0.01 mg/kg for both pesticides and was defined as the lowest concentration providing a signal-to-noise ratio (S/N) of ≥10. Accuracy and precision were evaluated by recovery experiments performed for each matrix at three fortification levels corresponding to the LOQ, 10 × LOQ, and a concentration corresponding to the Korean maximum residue limit (MRL). Five replicate analyses (*n* = 5) were conducted at each fortification level. Accuracy was expressed as the mean recovery (%), whereas precision was expressed as the relative standard deviation (RSD, %) of the replicate measurements. Matrix-matched calibration standards were used for each investigated matrix to compensate for potential matrix-induced signal suppression or enhancement during LC–MS/MS analysis. Calibration curves were constructed separately for each matrix, and linearity was evaluated using the coefficient of determination (*R*^2^). Although matrix effects were not independently quantified by comparing solvent-based and matrix-matched calibration slopes, the use of matrix-matched calibration was intended to minimize matrix-related quantification bias. The LOQ of 0.01 mg/kg was evaluated in each matrix based on a signal-to-noise ratio of at least 10 and recovery experiments performed at the LOQ fortification level using five replicate samples (*n* = 5).

### 2.7. Processing Factors

The processing factor (PF) is used to quantitatively evaluate changes in pesticide residue concentrations resulting from food processing [13,14]. PF is generally defined as the ratio of the pesticide residue concentration in the processed product to that in the corresponding starting material [13,14]. For milling fractions, PFs were calculated relative to whole grain, whereas PFs for subsequent processing steps, including cooking and rice-cake preparation, were calculated relative to the corresponding starting material (husked or polished rice). When the residue concentration in a processed product was below the LOQ, an upper-bound PF was calculated by dividing the LOQ by the residue concentration in the corresponding starting material [28]. PFs were not calculated when the residue concentration in the starting material was below the LOQ [13,28]. PFs were calculated using the following equation:

(1) $$\mathrm{PF}=\frac{\text{Residue concentration in processed product}\;(\mathrm{mg}/\mathrm{kg})}{\text{Residue concentration in corresponding starting material}\;(\mathrm{mg}/\mathrm{kg})}$$

### 2.8. Dietary Exposure Assessment Methodology

Dietary exposure to buprofezin and etofenprox was assessed for the edible rice fractions and their processed products. The assessment focused on chronic dietary exposure using mean pesticide residue concentrations and average daily food-consumption data. Daily food-consumption data were obtained from the Korea Health Industry Development Institute (KHIDI) based on the Korea National Health and Nutrition Examination Survey. The daily consumption amounts used for polished rice, husked rice, and rice cake were 117.30, 4.90, and 14.46 g/person/day, respectively [29]. In the absence of separate consumption data for cooked rice, the reported consumption amounts for polished rice and husked rice were used as proxy values for their cooked counterparts. This simplifying assumption allowed an illustrative comparison of dietary exposure across processing treatments. However, because rice absorbs water during cooking, equivalent weights of uncooked and cooked rice do not necessarily represent equivalent amounts of rice consumed. Therefore, comparisons of dietary exposure between uncooked and cooked rice should be interpreted with caution.

The estimated daily intake (EDI) of each pesticide was calculated using the mean residue concentration, daily food consumption, and an average adult body weight of 60 kg, as follows:

(2) $$\mathrm{EDI}\;(\mathrm{mg}/\mathrm{kg b}.w./\mathrm{day})=\frac{C\times F}{B.W.}$$

where *C* is the mean pesticide residue concentration (mg/kg), *F* is the daily consumption of the corresponding food (kg/day), and *B.W.* is the average body weight (60 kg). Dietary exposure was expressed as a percentage of the acceptable daily intake (%ADI) using the following equation:

(3) $$\%\mathrm{ADI}=\frac{\mathrm{EDI}}{\mathrm{ADI}}\times 100$$

The ADI values used for buprofezin and etofenprox were 0.009 and 0.03 mg/kg b.w./day, respectively, based on the evaluations of the Joint FAO/WHO Meeting on Pesticide Residues (JMPR) [30,31]. ADI values were used as the toxicological reference values because the assessment focused on chronic dietary exposure. For samples with quantifiable pesticide residues, EDI and %ADI were calculated using the measured mean residue concentrations. For samples with residue concentrations below the limit of quantification (LOQ; 0.01 mg/kg), an additional upper-bound exposure scenario was evaluated by substituting the LOQ for the unquantified residue concentration. The upper-bound EDI and %ADI were calculated using the same food-consumption amounts, body weight, and ADI values as those applied in the primary exposure assessment. These upper-bound estimates were reported separately from exposure estimates based on quantified residues. Hulls, inner chaff, and rice straw were excluded from the dietary exposure assessment because they were not considered direct human food commodities in this study.

### 2.9. Statistical Analysis

Pesticide residue concentrations were expressed as the mean ± standard deviation (SD) of three independent field replicates (*n* = 3). Statistical analyses were performed separately for buprofezin and etofenprox. Differences in residue concentrations among the five milling-related fractions (whole grain, husked rice, polished rice, hulls, and inner chaff) were evaluated using the Friedman test, accounting for the paired structure of samples derived from the same field replicate. Given the small sample size, exact permutation-based *p*-values were calculated for the Friedman test. PFs were calculated separately for each independent field replicate and summarized as mean ± SD to assess their variability. Samples with residue concentrations below the LOQ were excluded from quantitative statistical comparisons. Statistical significance was assessed at *p* < 0.05. Because only three independent field replicates were available, individual pairwise comparisons were not used to establish statistically significant differences between specific processing fractions. Statistical comparisons were performed separately for each pesticide because buprofezin and etofenprox were applied in different field plots under pesticide-specific treatment conditions. Direct statistical comparisons between pesticides were not performed. PFs were summarized descriptively as mean ± SD across the three independent field replicates, and no inferential comparisons of PFs among individual processing treatments were conducted.

## 3. Results and Discussion

### 3.1. Validation of the Analytical Method

**Figure 3:**
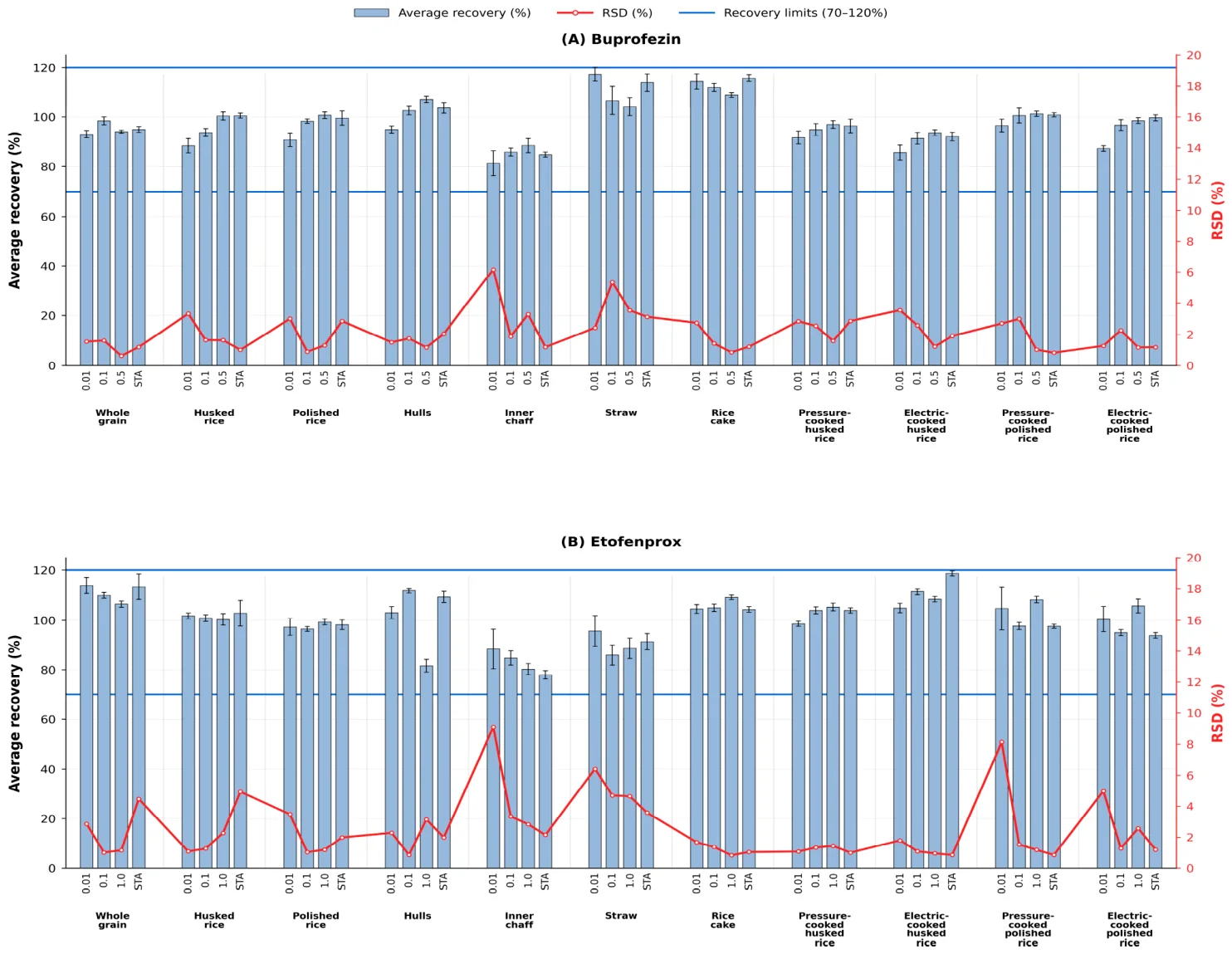
Mean recoveries and relative standard deviations (RSDs) of buprofezin (**A**) and etofenprox (**B**) in rice and processed rice products at different fortification levels. Error bars represent standard deviations (*n* = 5). Blue horizontal lines indicate the acceptable recovery range of 70–120%, and the red line represents RSD. STA, storage stability test at 0.1 mg/kg.

The analytical methods provided an LOQ of 0.01 mg/kg for both buprofezin and etofenprox in all investigated matrices, with signal-to-noise ratios of at least 10. The LOQ was further supported by recovery and precision evaluations at 0.01 mg/kg using five replicate fortified samples for each matrix. Although the magnitude of matrix effects was not quantified independently, matrix-specific calibration was employed to reduce potential bias associated with differences in matrix composition. Matrix-matched calibration curves showed good linearity over the range of 2–100 µg/L for whole grain, husked rice, polished rice, cooked rice, and rice cake, and 1–100 µg/L for straw, hulls, and inner chaff. The coefficients of determination (*R*^2^) exceeded 0.999 for all matrices (Table 2). Method accuracy and precision were assessed at three fortification levels corresponding to the LOQ, 10 × LOQ, and a concentration corresponding to the Korean MRL. Mean recoveries of buprofezin ranged from 81.3% to 117.3%, with relative standard deviations (RSDs) of 0.63–6.16%, whereas those of etofenprox ranged from 80.2% to 113.8%, with RSDs of 0.83–9.09% (Figure 3). These results met the method-performance criteria specified in CXG 90-2017 [27], indicating that the analytical methods were suitable for quantifying buprofezin and etofenprox in rice and the investigated processing fractions.

### 3.2. Results of Pesticide Residue Analysis

#### 3.2.1. Residue Concentrations of Pesticides in Whole Grain and Processed Products

**Figure 4:**
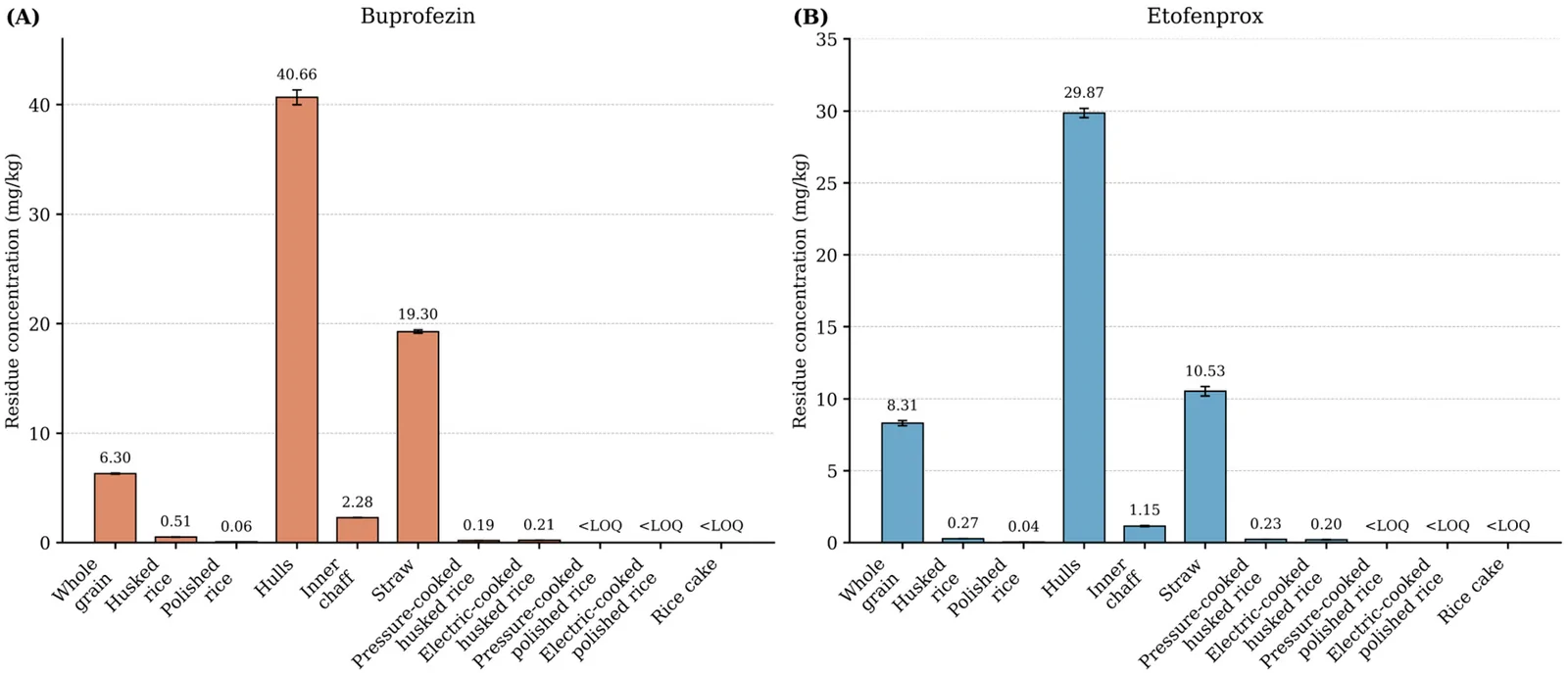
Mean residue concentrations of buprofezin (**A**) and etofenprox (**B**) in whole grain, straw, and processed rice products. Error bars represent standard deviations (*n* = 3). Values below the limit of quantification (<LOQ) are indicated accordingly.

Residue concentrations of buprofezin and etofenprox varied substantially among whole grain, milling fractions, and processed rice products (Figure 4). For buprofezin, residue concentrations were 6.30 mg/kg in whole grain, 40.66 mg/kg in hulls, 2.28 mg/kg in inner chaff, 0.51 mg/kg in husked rice, and 0.06 mg/kg in polished rice. The corresponding concentrations of etofenprox were 8.31, 29.87, 1.15, 0.27, and 0.04 mg/kg, respectively. Relative to whole grain, buprofezin residues decreased by 91.9% in husked rice and 99.0% in polished rice, whereas etofenprox residues decreased by 96.8% and 99.5%, respectively. These results demonstrate that milling markedly reduced the residues of both pesticides in the edible rice fractions. Statistical analysis further confirmed differences in pesticide residue concentrations among the five milling-related fractions. The Friedman test showed an overall difference in residue concentrations for both buprofezin and etofenprox (χ^2^ = 12.00, exact *p* < 0.001 for each pesticide). The ranking of residue concentrations was consistent across all three independent field replicates, following the order of hulls > whole grain > inner chaff > husked rice > polished rice. These findings indicate a consistent distribution pattern across the investigated milling fractions. However, because the analysis was based on only three independent field replicates, the statistical results should be interpreted cautiously, and significance between individual processing fractions was not established.

In contrast, hulls contained substantially higher residue concentrations than whole grain, while inner chaff contained higher concentrations than husked and polished rice. This distribution indicates that a large proportion of the residues present at harvest was associated with the outer portions of the grain. Similar heterogeneous residue distributions have been reported in field-treated rice, with residue concentrations changing as the outer grain layers are removed during processing [15,32]. Because the hull represents a relatively small fraction of the total grain mass, preferential association of residues with this fraction can result in higher concentrations on a unit-weight basis. The progressive removal of the hull and other outer grain layers therefore appears to be a major factor contributing to the lower residue concentrations observed in husked and polished rice.

The physicochemical properties of the two pesticides may also have influenced their distribution and behavior during processing. Buprofezin has a log P of approximately 4.93, whereas etofenprox is more hydrophobic, with a log P of approximately 6.9 and very low water solubility [16,18]. Physicochemical properties such as hydrophobicity and water solubility can affect the retention, redistribution, and removal of pesticide residues during food processing [11,13]. The relatively high hydrophobicity of both pesticides may have contributed to their association with the outer grain fractions, although this mechanism was not directly investigated in the present study [32,33]. However, residue distribution under field conditions is also influenced by factors such as spray deposition, penetration into plant tissues, formulation, environmental dissipation, and the interval between application and harvest [5,6,33,34].

Further reductions were observed during cooking. In husked rice, buprofezin decreased from 0.51 mg/kg before cooking to 0.19 mg/kg after pressure cooking and 0.21 mg/kg after electric cooking, corresponding to reductions of 62.7% and 58.8%, respectively. Etofenprox decreased from 0.27 mg/kg to 0.23 and 0.20 mg/kg, corresponding to reductions of 14.8% and 25.9%, respectively. Thus, the additional reduction during cooking was greater for buprofezin than for etofenprox under the conditions of this study. The greater hydrophobicity and lower water solubility of etofenprox may have contributed to its comparatively smaller reduction during washing and cooking [16,19]. Vapor pressure and thermal stability may also influence pesticide residue behavior during heating. Buprofezin has a higher vapor pressure (0.042 mPa at 20 °C) than etofenprox (8.13 × 10^−4^ mPa at 20 °C), suggesting differences in their potential for volatilization [18,19]. However, previous research has shown that pesticide residue reductions during rice cooking do not necessarily correlate with vapor pressure [7]. Thermal degradation may also contribute to residue reduction. A previous FAO evaluation reported that etofenprox was stable during storage at 80 °C but underwent partial degradation at 100 °C [35]. Nevertheless, these findings cannot be directly extrapolated to the cooking conditions of the present study, and thermal degradation was not independently quantified for either pesticide. Furthermore, because washing, soaking, water absorption, and heating occurred sequentially, the contributions of individual processing mechanisms could not be distinguished. Therefore, the observed differences between buprofezin and etofenprox cannot be attributed to any single physicochemical property or processing mechanism. Furthermore, changes in residue concentrations during processing may reflect both actual pesticide removal and changes in the mass or moisture content of the processed material. In particular, water absorption during cooking may contribute to lower residue concentrations expressed on a wet-weight basis through dilution. Because processing yields and moisture changes were not quantitatively determined for each processing stage, the relative contributions of pesticide removal and mass-related concentration changes could not be distinguished. Therefore, the reported residue reductions and PFs should be interpreted as changes in residue concentrations rather than direct measures of pesticide mass removal.

Previous studies have also reported reductions in buprofezin and etofenprox residues during rice processing. Buprofezin residues decreased during washing and cooking [36], while etofenprox residues progressively decreased during washing, soaking, grinding, and steaming in rice-cake production [16]. In the present study, the initial concentrations of buprofezin and etofenprox in polished rice were only 0.06 and 0.04 mg/kg, respectively, and both pesticides were below the LOQ after pressure and electric cooking. Consequently, quantitative comparison between the two cooking methods was not possible for polished rice.

Both pesticides were also below the LOQ in rice cake. The combined effects of washing, 12-h soaking, grinding, and steaming may have contributed to the further reduction in residues. Etofenprox residues have been reported to decrease progressively during rice-cake processing, with an overall reduction of 82.1% in high-residue samples [16], and similar reductions in buprofezin residues have also been observed during rice-cake processing [36]. The present study extends these findings by demonstrating residue reductions throughout sequential processing using rice obtained from pesticide applications under field conditions.

Rice straw contained relatively high residue concentrations, with 19.30 mg/kg of buprofezin and 10.53 mg/kg of etofenprox. These levels likely reflect the direct exposure of above-ground vegetative tissues during pesticide application. Pesticide residues in rice straw are of particular relevance because straw can be used as livestock feed [37]. Accordingly, although rice straw is not directly consumed as human food, residues in this fraction should be considered separately when evaluating the distribution of pesticide residues in rice plants and their potential entry into the livestock-feed chain.

#### 3.2.2. Processing Factor

**Figure 5:**
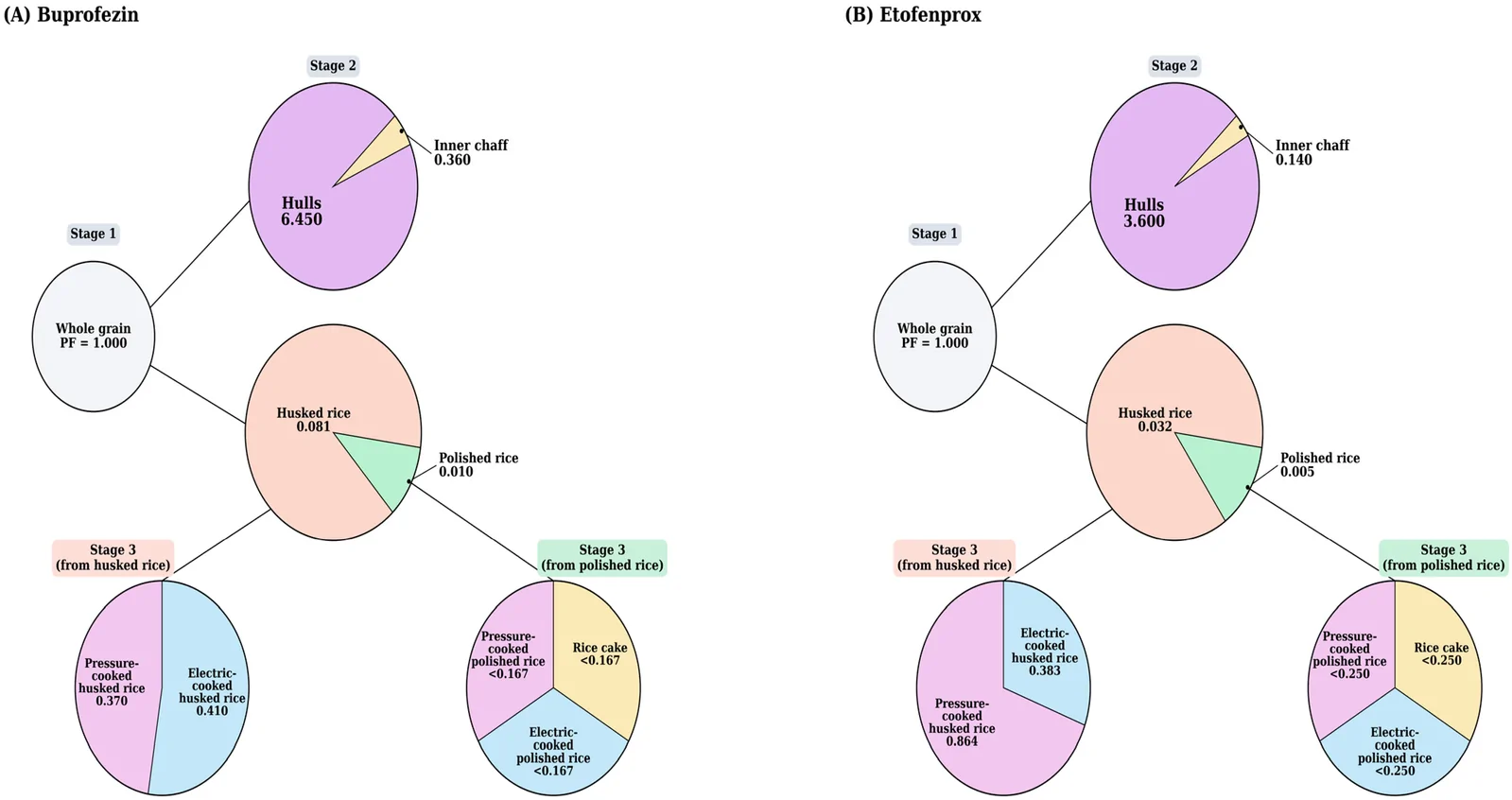
Schematic representation of the processing factors (PFs) of buprofezin (**A**) and etofenprox (**B**) across sequential rice-processing stages. PFs for hulls, inner chaff, husked rice, and polished rice were calculated relative to whole grain, whereas PFs for pressure- and electric-cooked husked rice were calculated relative to uncooked husked rice. For products derived from polished rice, including cooked polished rice and rice cake, residue concentrations were below the limit of quantification (LOQ; 0.01 mg/kg). Therefore, upper-bound PFs were calculated relative to uncooked polished rice by dividing the LOQ by its residue concentration.

PFs for buprofezin and etofenprox were calculated for each processing stage and are presented in Figure 5. For milling fractions, PFs were calculated relative to whole grain (PF = 1.00). The PFs of buprofezin and etofenprox were 6.45 and 3.60 in hulls, 0.360 and 0.140 in inner chaff, 0.081 and 0.032 in husked rice, and 0.010 and 0.005 in polished rice, respectively. Thus, PFs greater than 1 were observed only in hulls, whereas all edible rice fractions showed PFs substantially below 1. Similar patterns have been reported for cereal processing, with PFs below 1 in edible flour or semolina fractions and PFs above 1 in bran and other outer fractions [10,14]. These results demonstrate that PFs can vary markedly among milling fractions depending on the portion of the grain separated during processing. To assess variability among independent field replicates, PFs were calculated separately for each replicate and expressed as mean ± standard deviation (SD), as presented in Supplementary Table S1.

For subsequent cooking, PFs were calculated relative to uncooked husked rice to distinguish the effect of cooking from that of the preceding milling process. Accordingly, PFs for milling and cooking were expressed relative to their respective starting materials and should not be interpreted as values calculated on a common reference basis. Stage-specific PFs can be multiplied to obtain an overall PF only when the successive processing steps form a continuous pathway and the corresponding reference materials are consistent. The PFs of buprofezin were 0.373 and 0.412 for pressure- and electric-cooked husked rice, respectively, whereas those of etofenprox were 0.852 and 0.741, respectively. The lower cooking-stage PFs for buprofezin indicate a greater proportional reduction during cooking than for etofenprox. No consistent tendency toward lower PFs was observed for either pressure or electric cooking across the two pesticides.

For products prepared from polished rice, both pesticides were below the LOQ (<0.01 mg/kg) after pressure cooking, electric cooking, and rice-cake preparation. Therefore, exact PFs could not be determined. Instead, upper-bound PFs were calculated by dividing the LOQ by the residue concentration in uncooked polished rice, consistent with the approach used when residues in processed products are below the LOQ [28]. The resulting upper-bound PFs were <0.167 for buprofezin and <0.250 for etofenprox for both cooked polished rice and rice cake. Because these values represent upper bounds rather than measured PFs, the actual PFs may have been lower. These upper-bound PFs were presented only to illustrate the maximum possible PFs based on the LOQ and were not used in the subsequent dietary exposure assessment. As described in Section 2.8, EDI and %ADI values were not quantitatively calculated for products with residue concentrations below the LOQ.

The PFs obtained in this study were derived from rice treated at application rates exceeding three times the recommended Korean GAP rates. The use of exaggerated application rates was intended to ensure quantifiable residues for determining PFs, consistent with OECD Test Guideline No. 508 [25]. Applying these PFs to rice treated under normal GAP conditions assumes that proportional residue changes during processing remain approximately independent of the initial residue concentrations under comparable processing conditions. However, this assumption was not directly verified because only one application-rate regime was investigated. Therefore, the applicability of the derived PFs to rice treated at the recommended GAP rate should be interpreted with caution, particularly for processed products with residue concentrations below the LOQ.

### 3.3. Dietary Exposure Assessment

Dietary exposure estimates for buprofezin and etofenprox in rice and processed rice products are summarized in Table 3. Exposure was evaluated using the residue concentrations measured in each product and the food-consumption data described in Section 2.8.

For buprofezin, the mean residue concentrations in polished rice and husked rice were 0.06 and 0.51 mg/kg, respectively, corresponding to %ADI values of 1.303% and 0.463%. Although the residue concentration was higher in husked rice than in polished rice, dietary exposure was greater for polished rice because its daily consumption (117.30 g/person/day) was substantially higher than that of husked rice (4.90 g/person/day). This result demonstrates that dietary exposure is influenced by both residue concentration and food consumption. Following cooking, the %ADI values for electric and pressure-cooked husked rice decreased to 0.191% and 0.172%, respectively. Buprofezin residues in pressure- and electric-cooked polished rice and rice cake were below the LOQ; therefore, exposure estimates based on quantified residues could not be calculated for these products.

A similar pattern was observed for etofenprox. The mean residue concentrations in polished rice and husked rice were 0.04 and 0.27 mg/kg, respectively, corresponding to %ADI values of 0.261% and 0.074%. Despite the lower residue concentration in polished rice, its substantially greater consumption resulted in higher dietary exposure than that associated with husked rice. The %ADI values for electric and pressure-cooked husked rice were 0.054% and 0.063%, respectively. Etofenprox residues in pressure- and electric-cooked polished rice and rice cake were below the LOQ; therefore, exposure estimates based on quantified residues could not be calculated for these products.

For processed products with residue concentrations below the LOQ, an additional upper-bound exposure scenario was evaluated by substituting the LOQ (0.01 mg/kg) for the unquantified residue concentrations. Based on the consumption assumptions used in this study, the upper-bound %ADI values for pressure- and electric-cooked polished rice were 0.217% for buprofezin and 0.065% for etofenprox. The corresponding values for rice cake were 0.027% and 0.008%, respectively. These estimates were lower than the maximum %ADI values calculated from quantified residues (1.303% for buprofezin and 0.261% for etofenprox). Nevertheless, the upper-bound estimates represent hypothetical exposure scenarios rather than measured exposure levels. Furthermore, because the consumption amounts for uncooked rice were used as proxies for cooked rice, differences in food mass resulting from water absorption should be considered when interpreting exposure comparisons between uncooked and cooked products.

The reduction in dietary exposure following rice processing is consistent with previous studies showing that milling and subsequent household processing can reduce pesticide residues before consumption [15,38]. Because residue concentrations in processed foods can differ considerably from those in harvested commodities, the use of residue concentrations measured in foods as consumed provides a more representative basis for dietary exposure assessment.

The highest %ADI values observed in this study were 1.303% for buprofezin and 0.261% for etofenprox, both in polished rice. Notably, these values were obtained even though both pesticides were applied at three times the recommended Korean GAP rate to maintain quantifiable residues throughout the processing study. Nevertheless, all calculated %ADI values represented only a small proportion of the respective ADIs. These results indicate that chronic dietary exposure to buprofezin and etofenprox through the evaluated rice and processed rice products was low relative to their respective ADIs under the conditions of this study.

Several limitations should be considered when interpreting the dietary exposure estimates. Although rice samples were independently collected, processed, and analyzed from three replicate field plots per pesticide treatment, all samples originated from a single experimental location in Anseong, Republic of Korea. Therefore, the samples do not represent the geographical or production-related variability of rice commercially available in Korea. Furthermore, both pesticides were applied at three times the recommended Korean GAP rate to obtain quantifiable residues throughout processing. Consequently, the reported maximum %ADI values of 1.303% for buprofezin and 0.261% for etofenprox represent exposure estimates under an exaggerated application scenario rather than actual GAP-based exposure. Assuming a directly proportional relationship between pesticide application rate and residue concentration, the corresponding %ADI values at the recommended GAP rate would be approximately 0.434% and 0.087%, respectively. However, these values are hypothetical estimates rather than experimentally verified exposure levels, and proportionality between application rates and residue concentrations may not always hold [34,39]. The dietary exposure assessment was also based on mean residue concentrations, average food-consumption data, and an assumed adult body weight of 60 kg, without accounting for variations in food consumption, body weight, or exposure from other dietary sources. Therefore, although the estimated chronic dietary exposures were low relative to the corresponding ADIs under the investigated conditions, the results should not be generalized to the actual dietary exposure or health risks of the Korean population. Further assessments using geographically representative monitoring data and population-specific food-consumption information would be needed to characterize dietary risks more comprehensively.

## 4. Conclusions

This study evaluated changes in buprofezin and etofenprox residues throughout sequential rice processing and assessed their PFs and dietary exposure through rice and processed rice products. Milling markedly reduced pesticide residue concentrations in the edible rice fractions, with reductions of approximately 92–97% in husked rice and 99.0–99.5% in polished rice relative to whole grain. In contrast, higher residue concentrations were observed in hulls on a unit-weight basis, with PFs of 6.45 for buprofezin and 3.60 for etofenprox, consistent with greater retention of residues in the outer grain fractions. Cooking further reduced residues remaining after milling; in husked rice, buprofezin decreased by approximately 59–63% and etofenprox by approximately 15–26%, whereas both pesticides were below the LOQ (<0.01 mg/kg) in cooked polished rice and rice cake. The maximum %ADI values were 1.303% for buprofezin and 0.261% for etofenprox, both in polished rice. Although residue concentrations were lower in polished rice than in husked rice, its substantially greater consumption resulted in higher estimated dietary exposure, highlighting the importance of considering both residue concentration and food consumption in exposure assessment. The estimated dietary exposure levels were low relative to the respective ADIs under the conditions evaluated in this study. However, the field experiment was conducted using a single rice variety at one location with three independent field replicates, and the target pesticide application rates were set at three times the recommended Korean GAP rates to obtain quantifiable residues for processing analysis. The actual application rates were higher than the nominal threefold GAP rates but remained within the ±25% application-rate deviation considered in the FAO/JMPR guidance. Consequently, the processing factors and exposure estimates should not be directly generalized to rice produced under recommended GAP conditions without further validation. In addition, dietary exposure estimates were based on average consumption data and a standard adult body weight, while consumption amounts for uncooked rice were used as proxies for cooked rice. Although LOQ-based upper-bound exposure estimates were included for products with unquantifiable residues, these estimates depend on the specified assumptions and do not represent population-level upper-bound exposure. Despite these limitations, the results provide field-based information on changes in buprofezin and etofenprox residue concentrations during rice milling and subsequent processing, supporting the refinement of processing factors and dietary exposure assessments.

## Figures and Tables

**Table 1:** Detailed information of the test pesticides sprayed onto rice.

Site	Pesticide			Application Rate per Treatment			
		Korean GAP (kg a.i./ha)	3 × Korean GAP (kg a.i./ha)	Treatment	Active Ingredient (kg/ha)	Water (L/ha)	Active Ingredient (kg/hL)
Anseong	Buprofezin	0.192	0.576	1st	0.687	1908	0.0360
				2nd	0.674	1872	0.0360
				3rd	0.666	1850	0.0360
	Etofenprox	0.160	0.480	1st	0.585	1950	0.0300
				2nd	0.572	1950	0.0293
				3rd	0.583	1942	0.0300

**Table 2:** Coefficient of determination (*R*^2^) of buprofezin and etofenprox in different rice matrices.

Sample	Buprofezin		Etofenprox	
	Linear Range (µg/L)	*R* ^2^	Linear Range (µg/L)	*R* ^2^
**Straw**	1–100	0.9996	1–100	0.9998
**Hulls**	1–100	0.9999	1–100	0.9993
**Inner chaff**	1–100	0.9998	1–100	0.9991
**Whole grain**	2–100	0.9998	2–100	0.9996
**Husked rice**	2–100	0.9998	2–100	0.9999
**Polished rice**	2–100	0.9994	2–100	0.9996
**Rice cake**	2–100	0.9997	2–100	0.9999
**Husked rice cooked by pressure cooker**	2–100	0.9992	2–100	0.9999
**Husked rice cooked by electric cooker**	2–100	0.9993	2–100	0.9999
**Polished rice cooked by pressure cooker**	2–100	0.9994	2–100	0.9999
**Polished rice cooked by electric cooker**	2–100	0.9990	2–100	0.9999

**Table 3:** Dietary risk assessment of buprofezin and etofenprox in rice and processed rice products.

Pesticide	Sample	Mean Residue (mg/kg)	Food Daily Intake ^b^ (g)	EDI ^c^	ADI ^e^	%ADI ^f^
				mg/kg b.w./day		
**Buprofezin**	Polished rice	0.06	117.30	1.17 × 10^−4^	0.009	1.303
	Polished rice cooked by electric cooker	<0.01 ^a^		1.96 × 10^−5 d^		0.217 ^d^
	Polished rice cooked by pressure cooker	<0.01		1.96 × 10^−5 d^		0.217 ^d^
	Husked rice	0.51	4.90	4.17 × 10^−5^		0.463
	Husked rice cooked by electric cooker	0.21		1.72 × 10^−5^		0.191
	Husked rice cooked by pressure cooker	0.19		1.55 × 10^−5^		0.172
	Rice cake	<0.01	14.46	2.41 × 10^−6 d^		0.027 ^d^
**Etofenprox**	Polished rice	0.04	117.30	7.82 × 10^−5^	0.03	0.261
	Polished rice cooked by electric cooker	<0.01		1.96 × 10^−5 d^		0.065 ^d^
	Polished rice cooked by pressure cooker	<0.01		1.96 × 10^−5 d^		0.065 ^d^
	Husked rice	0.27	4.90	2.21 × 10^−5^		0.074
	Husked rice cooked by electric cooker	0.20		1.63 × 10^−5^		0.054
	Husked rice cooked by pressure cooker	0.23		1.88 × 10^−5^		0.063
	Rice cake	<0.01	14.46	2.41 × 10^−6 d^		0.008 ^d^

^a^ Less than the limit of quantification (LOQ), ^b^ Data from the Korea Health Industry Development Institute (2023), Korea, ^c^ Estimate daily intake calculated from the equation, residue (mg/kg) × food daily intake (kg/day)/body weight (60 kg), ^d^ Upper-bound exposure estimate calculated by substituting the LOQ (0.01 mg/kg) for residue concentrations below the LOQ. These values represent hypothetical upper limits under the specified consumption assumptions and are not based on quantified residues, ^e^ Acceptable daily intake, ^f^ (EDI/ADI) × 100.

## Data Availability

Data is contained within the article.

## Supplementary Materials

The following supporting information can be downloaded at: https://www.mdpi.com/article/10.3390/foods15213846/s1, Table S1: Processing factors (PFs) of buprofezin and etofenprox during rice milling and cooking, expressed as mean ± standard deviation (SD) of three independent field replicates (*n* = 3).
