# Supplementary material for: Residue Behavior, Processing Factors, and Dietary Exposure of Buprofezin and Etofenprox During Rice Processing
Source: Foods. 2026 Oct 27;15(21):3846. doi: 10.3390/foods15213846 (PMC13763478; doi:10.3390/foods15213846)
Supplement: Supplementary file 1 [file foods-15-03846-s001.zip › foods-4634812-supplementary.pdf]

Supplementary Materials

Residue Behavior, Processing Factors, and Dietary Exposure of Buprofezin and Etofenprox During Rice Processing

Dongju Kim [1], Seunghyeon Jo [2], Hyeonwoo Shin [3], Youngjin Ham [4], Junyoung Kim [5], Eunbeen Oh [6], Mihyun Cho [7], Moohyeog Im [7] and Keesung Kyung [1,*]

[1] Department of Environmental and Biological Chemistry, College of Agriculture, Life and Environment Sciences, Chungbuk National University, Cheongju 28644, Republic of Korea; kimdj6746@naver.com
[2] Republic of Korea Institute of Toxicology, Jeongeup 56212, Republic of Korea; seunghyeon.jo@kitox.re.kr
[3] NongHyup Chemical, Okcheon 29008, Republic of Korea; hyeonwoo@nhchemical.com
[4] Residue Study Division, AB Solution, Hwaseong 18514, Republic of Korea; youngjin0223@naver.com
[5] Republic of Korea Conformity Laboratories, Incheon 21999, Republic of Korea; tim8518@kcl.re.kr
[6] Dongbang Agro Corporation, Buyeo 33216, Republic of Korea; eboh01@dbagro.co.kr
[7] Department of Food Engineering, College of Engineering, Daegu University, Gyeongsan 38453, Republic of Korea; c_mh97@naver.com (M.C.); imh0119@daegu.ac.kr (M.I.)
* Correspondence: kskyung@cbnu.ac.kr; Tel.: +82-43-261-2562

Table S1. Processing factors (PFs) of buprofezin and etofenprox during rice milling and cooking, expressed as mean ± standard deviation (SD) of three independent field replicates (n = 3)

Processing stage	Buprofezin (mean ± SD)	Etofenprox (mean ± SD)
Milling fractions		
Whole grain (reference)	1.000	1.000
Hulls	6.454 ± 0.119	3.598 ± 0.179
Inner chaff	0.360 ± 0.007	0.139 ± 0.011
Husked rice	0.081 ± 0.002	0.032 ± 0.001
Polished rice	0.010 ± 0.000	0.005 ± 0.000
Cooking processes		
Pressure-cooked husked rice	0.373 ± 0.011	0.852 ± 0.050
Electric-cooked husked rice	0.410 ± 0.031	0.739 ± 0.053
Pressure-cooked polished rice	<0.167 [a]	<0.250 [a]
Electric-cooked polished rice	<0.167 [a]	<0.250 [a]
Rice cake	<0.167 [a]	<0.250 [a]

[a] Residue concentrations were below the limit of quantification (LOQ; 0.01 mg/kg). These values represent upper-bound PFs calculated by dividing the LOQ by the mean residue concentration in uncooked polished rice. Therefore, exact PFs and their standard deviations could not be determined.
